# Harnessing Natural Products to Surmount Drug Resistance in Gastric Cancer: Mechanisms and Therapeutic Perspectives

**DOI:** 10.7150/ijbs.113709

**Published:** 2025-07-11

**Authors:** QingShui Wang, Fangqin Xue, Yehuda G. Assaraf, Yao Lin

**Affiliations:** 1 Affiliated People's Hospital, Fujian-Macao Science and Technology Cooperation Base of Traditional Chinese Medicine-Oriented Chronic Disease Prevention and Treatment, Fujian-Hong Kong-Macau-Taiwan Collaborative Laboratory for the Inheritance and Innovation of Traditional Chinese Medicine, Fujian University of Traditional Chinese Medicine, Fuzhou 350122, Fujian, China.; 2 The Fred Wyszkowski Cancer Research Laboratory, Faculty of Biology, The Technion-Israel Institute of Technology, Haifa 3200003, Israel.; 3 Department of Gastrointestinal Surgery, Shengli Clinical Medical College of Fujian Medical University, Fujian Provincial Hospital, Fuzhou University Affiliated Provincial Hospital, Fuzhou, 350001, China.

**Keywords:** gastric cancer, chemotherapy resistance, natural products, overcoming chemoresistance, immunotherapy

## Abstract

Gastric cancer remains a major cause of mortality. The current standard of care of gastric cancer is based upon combination chemotherapy comprising platinum, fluoropyrimidines drugs and docetaxel. However, the efficacy of chemotherapy is hindered by intrinsic and acquired drug resistance, resulting in poor patient prognosis. Studies are currently exploring the potential of immunotherapy and other biologically targeted agents in the perioperative setting. To select the most efficacious therapy for advanced gastric cancer, including adenocarcinoma of the esophago-gastric junction, it is essential to determine key biomarkers including for example human epidermal growth factor receptor 2 (HER2) expression, programmed death-ligand 1 (PD-L1) combined positive score (CPS), Claudin 18.2, and microsatellite instability (MSI). Moreover, recent studies have highlighted the potential of natural products as effective agents in surmounting anticancer drug resistance in gastric cancer. These bioactive compounds exhibit various antitumor activities, including induction of apoptosis, inhibition of cell proliferation, modulation of autophagy, and most importantly reversal of distinct multidrug resistance (MDR) modalities. The present review provides a comprehensive analysis of the mechanisms underlying chemoresistance in gastric cancer and explores how natural products can overcome distinct MDR mechanisms. We further discuss the therapeutic potential of combining natural products with established chemotherapeutics and immunotherapy agents to enhance treatment efficacy and reduce adverse effects.

## 1. Introduction

Gastric cancer is a serious global health issue characterized by high incidence and mortality rates. It is estimated that over 1 million new cases of gastric cancer are diagnosed worldwide each year, making it one of the most common cancers globally^1^. Despite a general decline in incidence and mortality rates over the past few decades, gastric cancer remains the third leading cause of cancer-related deaths, particularly in East Asia, where nearly half of all cases occur^2^. The global burden of gastric cancer is influenced by various factors, including demographic changes, lifestyle choices, and the prevalence of risk factors such as *Helicobacter pylori* infection, smoking, and high-sodium diets^3^. While the age-standardized incidence and mortality rates have declined, the burden of gastric cancer is expected to increase in the coming years due to population growth and aging^4^, ^5^. These trends underscore the urgent need for improved strategies in gastric cancer management and treatment.

Chemoresistance of gastric cancer presents a significant challenge in the treatment and management of this disease^6^, ^7^. Despite advancements in chemotherapy, many patients display intrinsic or acquired resistance to treatment, which severely limits the effectiveness of therapeutic interventions. One of the key factors contributing to chemoresistance in gastric cancer is the upregulation of specific efflux pumps of the ATP-binding cassette (ABC) transporter superfamily which mediate drug extrusion, thereby markedly reducing the intracellular concentration of chemotherapeutic agents^8^, ^9^. Moreover, gastric cancers are highly enriched in hypoxic niches, and the degree of hypoxia is strongly correlated with the dismal prognosis of gastric cancer patients^10^. In this respect, stemness and chemoresistance in gastric cancer are the two root causes of poor patient outcomes. In this context, exploring alternative therapeutic avenues becomes crucial for enhancing patient outcomes.

Natural products have emerged as a promising therapeutic avenue towards the overcoming of drug resistance in gastric cancer. Recent studies have highlighted the multifaceted roles of natural products in combating gastric cancer^11^-^14^. These natural compounds exhibit multicomponent and multitarget characteristics, rendering them suitable candidates for innovative therapeutic strategies^15^. Studies have shown that various natural products can impede the progression of gastric cancer by modulating critical signaling pathways involved in tumor progression^16^-^19^. The incorporation of natural products into therapeutic regimens not only offers a complementary approach to conventional treatments but also addresses issues of drug resistance reversal and untoward toxicity commonly associated with chemotherapy. Therefore, further investigation into natural products may pave the way for novel therapies to surmount chemoresistance in gastric cancer.

## 2. Intrinsic and Acquired Mechanisms of Chemotherapy Resistance in Gastric Cancer

Gastric cancer frequently develops chemoresistance, presenting significant impediments to efficacious treatment. Deciphering the intrinsic and acquired mechanisms underlying gastric cancer resistance is critical for improving therapeutic outcomes.

### 2.1 Alterations in cell death pathways

A pivotal mechanism by which gastric cancer cells acquire chemotherapy resistance involves alterations in cell death pathways, particularly apoptosis and autophagy^20^. These changes enable cancer cells to evade the cytotoxic effects of chemotherapy.

#### 2.1.1 Apoptosis evasion

The complex biology and significant heterogeneity of gastric cancer complicate effective treatment strategies. A crucial factor in chemotherapy resistance is the evasion of apoptosis-a programmed cell death process essential for eliminating damaged or unwanted cells^21^. Dysregulation of apoptotic pathways allows cancer cells to survive despite exposure to chemotherapeutic agents^22^. One prominent player in apoptosis resistance is the overexpression of anti-apoptotic proteins, such as myeloid cell leukemia-1 (Mcl1), a member of the Bcl-2 family that promotes cell survival. Studies have indicated that myeloid cell leukemia-1 (Mcl1) is frequently overexpressed in gastric cancer cell lines, leading to a reduced sensitivity to chemotherapeutic drugs like cisplatin and 5-fluorouracil (5-FU)^23^. Furthermore, Mcl-1 protein levels were markedly increased in gastric cancer tissues than in the corresponding normal tissues^24^. This higher Mcl1 expression was correlated with T classification, metastasis, clinical stage, and venous invasion. Consequently, patients with higher Mcl1 levels displayed a poorer 5-year overall survival than patients with low Mcl1 levels. Univariate and multivariate analyses further suggested that Mcl1 overexpression was an independent prognostic marker for gastric cancer. Likewise, Mcl1 expression was detected in 127/182 (69.8%) gastric carcinoma patients^25^; Mcl1 was detected more frequently in the undifferentiated type and in the advanced stage of the disease. The prognosis of patients harboring Mcl1-positive tumors was significantly more dismal than those with an Mcl1-negative tumor. Multivariate analysis revealed that Mcl1 expression was an independent prognostic factor, as were lymph node metastases and tumor size. Hence, suppressing Mcl1 expression has been shown to enhance apoptosis in gastric cancer cells, suggesting that targeting this anti-apoptotic protein is a promising strategy to overcome chemotherapy resistance^23^. Moreover, alterations in the expression of pro-apoptotic and anti-apoptotic proteins, along with mutations in key signaling pathways, contribute to the observed resistance in gastric cancer. For instance, the activation of the Mitogen-Activated Protein Kinase (MAPK) and Protein Kinase B/mammalian Target of Rapamycin (AKT/mTOR) pathways has been implicated in promoting cell survival and resistance to drug-induced apoptosis^26^, ^27^. These pathways can be hijacked by cancer cells to evade the cytotoxic activity exerted by chemotherapeutics, leading to poorer treatment outcomes.

#### 2.1.2 Autophagy

Autophagy plays a critical dual role in cancer, particularly concerning chemotherapy resistance^28^, ^29^. In gastric cancer, autophagy can undergo upregulation in response to chemotherapeutic agents, allowing cancer cells to survive and recover from treatment. This phenomenon has been observed in studies where drug-resistant cancer cells exhibited autophagy as a survival mechanism following exposure to clinically relevant chemotherapeutics like 5-FU and cisplatin^30^. The activation of autophagy in these cells is associated with a morphology resembling type II programmed cell death, distinct from apoptosis^30^. Moreover, studies have shown that specific inhibition of autophagy can enhance the efficacy of chemotherapy. Targeting autophagy-related proteins such as Beclin 1 (BECN1) and Autophagy Related 7 (ATG7) has significantly increased the cytotoxic effects of 5-FU in drug resistant cell lines^30^. This suggests that autophagy not only contributes to the survival of cancer cells but also plays a pivotal role in the development of acquired resistance to chemotherapy.

In gastric cancer patients, a gene expression signature associated with acquired resistance to cisplatin and 5-FU has been identified, highlighting the involvement of the AKT/mTOR signaling pathway, which is closely linked to autophagy regulation^31^. The upregulation of genes within this pathway, such as AKT1 and ribosomal protein S6 (RPS6), indicates that autophagy may facilitate the survival of cancer cells under chemotherapeutic stress, thereby contributing to treatment failure^31^, ^32^.

#### 2.1.3 Other cell death pathways

In addition to apoptosis and autophagy, there are eleven distinct cell death pathways, including endolytic cell death, necroptosis, ferroptosis, reticulocyte death, pyroptosis, parthanatos, cuproptosis, lysosome-dependent cell death, oxidative death, anoikis, and alkaline death^33^. Among these pathways, ferroptosis and cuproptosis have been specifically linked to chemotherapy resistance in gastric cancer^34^, ^35^. One pivotal study demonstrated that activating ferroptosis can alleviate cisplatin resistance in gastric cancer cells by inhibiting the Nuclear factor erythroid 2-related factor 2/Kelch-like ECH-associated protein 1/Cystine/glutamate transporter (Nrf2/Keap1/xCT) signaling pathway. This finding suggests that targeting the ferroptotsis pathway may represent a novel strategy to enhance the effectiveness of chemotherapy^36^. Cuproptosis, a newly identified form of cell death that is dependent on copper levels, has garnered significant attention in the context of chemotherapy resistance, particularly in gastric cancer^37^, ^38^. The administration of copper ionophores has shown promise in inducing cuproptosis in cancer cells, potentially reversing resistance to chemotherapeutic agents such as cisplatin and doxorubicin^39^, ^40^.

### 2.2 Tumor Microenvironment (TME)

The TME is a complex and dynamic ecosystem comprising cancer cells, stromal cells, extracellular matrix (ECM), vasculature, and immune cells^41^. It plays a crucial role in chemotherapy resistance in gastric cancer by forming a protective niche that fosters tumor growth and survival under treatment-induced stress^42^, ^43^. It consists of various cellular and non-cellular components that interact through intricate signaling networks, providing biochemical and mechanical cues that drive tumor progression and therapeutic resistance^44^. Hypoxia within the TME drives tumor cell adaptation through activation of hypoxia-inducible factors (HIFs), leading to increased angiogenesis, metabolic reprogramming, and reduced drug delivery to cancer cells^45^. The ECM not only provides physical protection against drug penetration but also transduces mechanical and biochemical signals to cancer cells through integrin and receptor-mediated pathways, promoting survival and chemoresistance^46^. Cancer-associated fibroblasts (CAFs), a prominent stromal component, secrete growth factors, cytokines, and ECM proteins, further enhancing resistance through paracrine signaling and modulation of tumor cell behavior^47^. Immune cells within the TME, particularly tumor-associated macrophages (TAMs), often adopt a pro-tumorigenic M2 phenotype, secreting anti-inflammatory cytokines, suppressing anti-tumor immune responses, and directly or indirectly promoting drug resistance by supporting cancer cell survival and proliferation^48^. Furthermore, intercellular communication mechanisms, such as exosome-mediated transfer of resistance-associated molecules between stromal and tumor cells, facilitate the propagation of resistant phenotypes across the tumor^49^, ^50^.

#### 2.2.1 Hypoxia

Hypoxia is a common and critical feature of the TME in gastric cancer, primarily driven by inadequate blood supply and the resulting deprivation of oxygen^51^-^55^. Under hypoxic conditions, HIFs become stabilized and activate a complex transcriptional network that regulates genes involved in angiogenesis, metabolic reprogramming, cell survival, and the maintenance of cancer stemness. These cellular adaptations to hypoxia increase the expression and activity of ATP-binding cassette (ABC) drug efflux transporters, enhance DNA repair capacity, and modulate apoptotic signaling pathways, together contributing to both intrinsic and acquired resistance to chemotherapy^51^, ^56^. To address these challenges, various therapeutic strategies targeting hypoxia have been developed, including hypoxia-activated prodrugs, inhibitors of the HIF signaling pathway, and nanoparticle-based drug delivery systems. These approaches have shown promising efficacy in preclinical models by improving drug delivery to hypoxic tumor regions and enhancing treatment outcomes^57^-^58^, ^59^.

#### 2.2.2. Extracellular Matrix (ECM)

The ECM is another critical component of the TME, providing structural support and biochemical signals that regulate cancer cell behavior^60^. In gastric cancer, alterations in the composition and organization of the ECM can promote a chemoresistance phenotype. For instance, changes in ECM composition and stiffness can activate signaling pathways that enhance cell survival and proliferation, thereby contributing to resistance against antitumor agents^57^, ^61^**.**

Furthermore, the ECM can sequester drugs, reducing their availability to cancer cells. This phenomenon is particularly relevant in gastric cancer, where a dense ECM can act as a barrier to drug penetration, limiting the efficacy of chemotherapy^62^. Additionally, the ECM influences the tumor's response to treatment by inducing the secretion of various inflammatory cytokines from cells in the TME. These cytokines create a pro-tumorigenic microenvironment that supports cancer cell survival and proliferation, ultimately leading to cancer progression and therapy resistance^63^.

The interplay between the ECM and cancer cells is complex; cancer cells can also modify the ECM through the secretion of proteolytic enzymes and other factors. This reciprocal relationship not only facilitates tumor progression but also contributes to the establishment of a chemoresistance phenotype^64^.

#### 2.2.3. Cancer-Associated Fibroblasts (CAFs)

CAFs are increasingly recognized as key contributors to chemotherapy resistance in gastric cancer^65^, ^66^. They actively promote tumor progression by secreting growth factors, cytokines, and ECM components, which enhance cancer cell proliferation, survival, and migration^67^. CAF-mediated pro-survival signaling in cancer cells reduces their susceptibility to chemotherapeutics^68^. Specific CAF subpopulations, such as those expressing cyclophilin C (CypC), have been linked to poor prognosis and chemoresistance. CypC, a secreted enzyme facilitating protein folding, is associated with larger tumors, advanced disease stages, enhanced glucose metabolism, and ECM remodeling, contributing to tumor aggressiveness and resistance^69^, ^70^.

CAFs also modulate the TME to create a protective niche, shielding cancer cells from chemotherapy^71^. They influence drug transporter and metabolic enzyme expression, further altering drug efficacy^72^.

#### 2.2.4. Tumor-infiltrating immune cells

In addition to CAFs, tumor-infiltrating immune cells are critical players in the TME, contributing to chemoresistance in gastric cancer^73^, ^74^. These immune cells can either promote or inhibit tumor progression and response to therapy. Key immune cells include T lymphocytes, macrophages, dendritic cells, and myeloid-derived suppressor cells (MDSCs). In gastric cancer, the presence of regulatory T cells (Tregs) and MDSCs has been associated with an immunosuppressive environment that facilitates tumor growth and resistance to chemotherapy^75^, ^76^.

Macrophages in the TME can adopt different phenotypes; M2-polarized macrophages often promote tumor progression and chemoresistance via the secretion of anti-inflammatory cytokines and growth factors^77^. This polarization is influenced by various factors within the TME, including hypoxia and specific cytokines. Furthermore, the interaction between cancer cells and immune cells leads to the secretion of factors that enhance tumor cell survival, contributing to acquired resistance.

The dynamic interplay between tumor cells and the immune microenvironment is crucial for understanding drug resistance mechanisms. For instance, cancer cells can exploit immune checkpoints to evade immune surveillance, resulting in a more aggressive disease state^78^. Additionally, the TME can alter the expression of drug transporters and metabolic pathways in cancer cells, further compromising the response to chemotherapy. Recent studies highlight the importance of targeting the TME to overcome chemotherapy resistance^79^, ^80^. Strategies that aim to reprogram the immune landscape, such as immune checkpoint inhibitors or therapies that enhance cytotoxic T cell activity, are being explored as potential adjuncts to conventional chemotherapy^81^.

#### 2.2.5. Tumor Cell-TME interactions via cytokines and exosomes

Cytokines and exosomes are key mediators of communication within the TME, facilitating the exchange of signals between cancer cells and surrounding stromal cells. This intercellular communication significantly contributes to chemotherapy resistance.

Tumor-derived exosomes (TDEs) are small extracellular vesicles that carry bioactive molecules, including proteins, lipids, and nucleic acids, which can modulate the behavior of recipient cells in the TME^82^. These exosomes can induce phenotypic changes in stromal cells, such as fibroblasts and immune cells including TAMs, thereby promoting a supportive environment for tumor growth and drug resistance^83^. Apart from the critical roles of TDEs in tumor progression, TDEs also contribute to drug resistance and attenuate the effective response to antitumor immunotherapy. Mounting evidence indicates that TDEs contain a large number of nucleic acids that may transform drug-sensitive cancer cells to a multidrug resistance phenotype. Specifically, TDEs deliver P-glycoprotein (P-gp), an ATP-dependent multidrug transporter, in an autocrine manner to induce the ATP-driven expulsion of cytotoxic drugs.

Exosomes released by gastric cancer cells can carry specific miRNAs that influence the expression of genes involved in drug resistance pathways^84^, ^85^. For instance, exosomal miRNAs can target anti-apoptotic factors, enhancing the survival of cancer cells under chemotherapeutic cytotoxic stress^83^. Moreover, the TME secretes various cytokines that alter cancer cell behavior and their interactions with the stroma. Cytokines such as hepatocyte growth factor (HGF) have been shown to mediate resistance to targeted therapies in a paracrine manner, highlighting the importance of soluble factors in the TME^86^. Moreover, CAFs within the TME contribute to chemotherapy resistance by secreting factors that promote tumor cell survival and proliferation. The interplay between CAFs and gastric cancer cells leads to metabolic reprogramming and ECM remodeling, which are critical factors for maintaining a drug-resistance phenotype^87^. Understanding these interactions is essential for developing novel therapeutic strategies aimed at surmounting drug resistance in gastric cancer.

#### 2.2.6. Intercellular communication

Intercellular communication within the TME involves a complex network that facilitates signal exchange between cancer cells and their surroundings^88^. This communication occurs through direct cell-to-cell contact or via soluble factors like cytokines, growth factors, and extracellular vesicles. CAFs and TAMs are key players, often secreting factors that promote tumor survival and drug resistance. Recent studies have demonstrated that CAFs enhance the resistance of gastric cancer cells to chemotherapeutic agents by secreting growth factors such as HGF and transforming growth factor-β (TGF-β)^89^. These factors activate signaling pathways that promote tumor cell survival and proliferation, counteracting the cytotoxic effects of chemotherapy.

The role of exosomes in intercellular communication has also gained attention. Exosomes can transfer proteins, lipids, and RNA between cells, facilitating the transfer and spread of resistance mechanisms^90^. For example, exosomal microRNAs from resistant gastric cancer cells can be taken up by neighboring drug sensitive cells, leading to altered gene expression and enhanced chemotresistance^91^, ^92^.

### 2.3 LncRNAs in therapy resistance

Long non-coding RNAs (LncRNAs) play a critical role in mediating resistance to cancer therapies^93^-^96^. LncRNAs have emerged as critical regulators of gene expression and cellular processes that impact tumor behavior, including drug resistance^97^. Dysregulation of LncRNAs can lead to altered signaling pathways that promote cancer cell survival and proliferation, thereby contributing to resistance against chemotherapeutic agents^98^-^100^. For instance, specific LncRNAs have been shown to modulate the expression of genes involved in drug metabolism, apoptosis, and the TME, which are pivotal in determining the sensitivity of gastric cancer cells to chemotherapy^101^. Furthermore, LncRNAs can interact with miRNAs and proteins, forming complex regulatory networks that complicate the treatment landscape^102^.

In immunotherapy, LncRNAs affect immune checkpoints and cytokine expression, influencing therapeutic efficacy^103^, ^104^. Understanding LncRNAs involved in these processes may provide new therapeutic targets to enhance treatment effectiveness and overcome resistance^105^. Furthermore, the interplay between LncRNAs, miRNAs, and circular RNAs contributes to resistance by regulating transporters and apoptosis, complicating drug resistance mechanisms in gastric cancer^106^.

### 2.4 Epigenetic modifications

Epigenetic modifications play a crucial role in the development of chemotherapy resistance in gastric cancer^107^. These modifications can be broadly categorized into several types, including DNA methylation and histone modifications.

#### 2.4.1 DNA methylation

Epigenetic modifications, particularly DNA methylation, are crucial in the development of chemotherapy resistance in gastric cancer^108^. These modifications can lead to the silencing of tumor suppressor genes and the activation of oncogenes, thereby contributing to the malignant phenotype of cancer cells 109. In gastric cancer, aberrant DNA methylation patterns have been identified, influencing gene expression and affecting the response to chemotherapy^110^. Recent studies have shown that specific genes involved in drug metabolism and DNA repair pathways are often subject to epigenetic regulation through methylation. For example, methylation of the promoter regions of genes such as MutL Homolog 1 (MLH1), which is involved in DNA mismatch repair, has been associated with resistance to certain chemotherapeutic agents. This silencing can result in the accumulation of DNA damage ultimately leading to treatment failure^26^. Mutations in the MLH1 gene are linked to microsatellite instability (MSI) found in hereditary nonpolyposis colon cancer. Moreover, the interplay between DNA methylation and other epigenetic modifications including histone modifications and non-coding RNAs, further complicates the landscape of chemotherapy resistance. The dysregulation of these epigenetic mechanisms can generate a feedback loop that enhances the survival of cancer cells under therapeutic cytotoxic stress. For example, upregulation of certain microRNAs can lead to the downregulation of DNA repair genes, thereby promoting resistance to DNA-damaging agents^111^.

Deciphering the role of epigenetic modifications in chemotherapy resistance not only elucidates the molecular mechanisms underlying gastric cancer but may also pave the way towards potential avenues for therapeutic interventions. Targeting the epigenetic landscape of cancer cells through the use of demethylating agents or histone deacetylase inhibitors may restore the expression of silenced tumor suppressor genes and enhance the efficacy of existing chemotherapeutic regimens^112^-^114^.

#### 2.4.2 Histone modifications

Histone modifications are essential for regulating gene expression in gastric cancer cells^115^, ^116^. These modifications can promote or inhibit gene transcription, depending on the specific type of modification and the context in which it occurs. For instance, histone acetylation is generally associated with gene activation, while histone methylation can lead to either activation or repression depending on the specific histone residue modified^117^. In the context of chemotherapy resistance, aberrant histone modifications can silence tumor suppressor genes and activate oncogenes, contributing to the malignant phenotype of gastric cancer cells. For example, hypermethylation of histone H3 at lysine 27 (H3K27me3) has been linked to the repression of genes critical for apoptosis and drug sensitivity^118^. This epigenetic alteration can enable cancer cells to survive in the presence of chemotherapeutic agents, thereby facilitating the emergence of drug resistance.

Moreover, the interplay between histone modifications and ncRNAs has emerged as a significant area of research. NcRNAs modulate the activity of histone-modifying enzymes, leading to changes in the epigenetic landscape of gastric cancer cells^119^.

### 2.5. Epithelial-Mesenchymal Transition (EMT)

EMT is a central biological process that enables epithelial cells to acquire mesenchymal characteristics, enhancing their migratory and invasive capabilities^120^. This transition is not only pivotal during normal embryonic development but also plays a significant role in cancer progression and metastasis. In gastric cancer, aberrant activation of EMT has been linked to increased tumor aggressiveness and poor clinical outcomes^121^, ^122^. The EMT process is characterized by the loss of epithelial markers, such as E-cadherin, and the gain of mesenchymal cell surface markers, including N-cadherin, and syndecan-1, cytoskeletal markers vimentin, β-catenin and FSP-1 as well as the transcription factors snail1, snail 2, LEF-1 and Ets-1^123^, ^124^.

Recent studies have highlighted a strong association between EMT and chemotherapy resistance in gastric cancer. Activation of EMT-related signaling pathways can lead to enhanced drug efflux, increased DNA repair capabilities, and evasion of apoptosis, all of which contribute to chemotherapy resistance^125^, ^126^. For instance, upregulation of ABC efflux transporters during EMT can result in the extrusion of multiple chemotherapeutic agents, markedly reducing their intracellular concentrations and cytotoxic activity^127^-^129^. Additionally, the mesenchymal phenotype is often associated with a more resilient cellular state that can withstand the cytotoxicity exerted by chemotherapeutics^130^.

Unraveling the molecular mechanisms underlying EMT and its role in drug resistance is crucial for developing novel therapeutic strategies. Targeting the pathways involved in EMT may provide opportunities to sensitize gastric cancer cells to chemotherapy and improve treatment outcomes. For example, inhibiting key transcription factors that drive EMT such as Snail Family Transcriptional Repressor 1 (SNAI1), and Zinc Finger E-Box Binding Homeobox 1 (ZEB1) could reverse the mesenchymal phenotype and restore sensitivity to chemotherapeutic agents^131^. Furthermore, integrating therapies that target both the tumor cells and the TME may enhance the efficacy of existing treatments and overcome the challenges posed by EMT-dependent drug resistance^132^.

The multifaceted mechanisms of chemoresistance in gastric cancer, ranging from alterations in cell death pathways and the tumor microenvironment to epigenetic modifications, highlight the complexity of this disease. Overcoming these challenges necessitates innovative therapeutic strategies, such as the utilization of natural products, which have demonstrated promising potential in circumventing drug resistance and enhancing treatment efficacy.

## Natural Products for Overcoming Drug Resistance in Gastric Cancer

As the mechanisms underlying chemoresistance in gastric cancer are increasingly deciphered, there is a growing need in exploring novel therapeutic strategies that can effectively combat this major impediment. Natural products, with their diverse chemical structures and biological activities, have historically played a significant role in drug discovery and development^133^. These bioactive compounds, derived from plants, microorganisms, and marine organisms, have shown potential in enhancing the efficacy of existing chemotherapeutics and overcoming drug resistance mechanisms. Recent studies have highlighted the ability of various natural products to modulate key pathways involved in drug resistance, including inhibition of tumor cell survival pathways, modulation of the TME, and targeting cancer stem cells^134^-^136^. Additionally, their capacity to act as chemosensitizers when used in combination with conventional chemotherapy offers a promising avenue for improving treatment outcomes in gastric cancer.

### 3.1. Targeting cancer stem cells

Natural products have gained attention for their capacity to target CSCs and reverse drug resistance. Several studies have identified compounds that exhibit significant anti-CSC activities by modulating key signaling pathways involved in CSC maintenance and survival.

#### 3.1.1 Single compounds

Evodiamine, derived from the traditional herbal medicine *Tetradium ruticarpum* (*Evodia rutaecarpa*), has been recognized as an inhibitor of the Wnt/β-catenin signaling pathway, which is essential for CSC self-renewal and maintenance^137^. Evodiamine has been shown to inhibit the proliferation of gastric cancer stem cells (GCSCs) and induce apoptosis. In addition, evodiamine reduces the sphere-forming ability of these cells and decreases the expression of pluripotency factors such as Sex Determining Region Y-Box 2 (SOX2), Kruppel-Like Factor 4 (KLF4), B-cell lymphoma-2-related protein A1 (BMI1), and Octamer-Binding Transcription Factor 4 (OCT4). Moreover, evodiamine suppresses EMT markers, including Slug (SNAI2), ZEB1, and vimentin, which are associated with metastasis and chemoresistance. By targeting the Wnt/β-catenin pathway, evodiamine effectively diminishes the stemness and invasive properties of GCSCs^138^.

Sulforaphane, a naturally occurring compound in broccoli and broccoli sprouts, has also shown promising results in targeting GCSCs^139^. It significantly reduces tumor sphere formation and decreases the expression of CSC markers. Sulforaphane exerts its inhibitory effects by inducing apoptosis and suppressing proliferation of GCSCs. The underlying mechanism involves inhibition of the Sonic Hedgehog (SHH) signaling pathway, a key regulator of CSC maintenance and stemness^140^. By suppressing this pathway, sulforaphane effectively reduces the stem-like characteristics of GCSCs, highlighting its potential as a therapeutic agent in gastric cancer intervention^140^.

Cyclophilin A (CypA) inhibitors, such as compound 9 (C9) and cyclosporin A (CSA), have been explored for their activity against GCSCs. These inhibitors suppress proliferation and induce apoptosis of GCSCs by regulating the CypA/CD147-mediated AKT and MAPK signaling pathways^141^. Treatment with C9 and CSA led to cell cycle arrest at the G_0_/G_1_ phase and activation of the caspase cascade. Furthermore, these compounds significantly decreased the expression of key CSC markers, including CD133, CD44, Integrin Subunit Alpha 6 (ITGA6), SOX2, OCT4, and Nanog. These findings suggest that targeting the CypA/CD147 axis may be an effective strategy to eradicate GCSCs^141^.

Isoliquiritigenin (ISL), a bioactive flavonoid found in *Helichrysum petiolare* (i.e., licorice plant), has demonstrated the ability to inhibit gastric cancer stemness and modulate the TME^142^. ISL downregulates glucose-regulated protein 78 (GRP78/BiP), a major unfolded protein response (UPR) regulator, an endoplasmic reticulum (ER) chaperone protein critical for protein quality control in the ER, as well as controlling the activation of the ER-transmembrane signaling molecules. Hence, GRP78/BiP is well documented as an ER stress marker 51. GRP78 has been implicated in chemoresistance and maintenance of CSC characteristics. By suppressing GRP78, ISL reduces stemness-related protein expression, inhibits CSC-like properties, and decreases cancer-associated fibroblast activation. In xenograft models, ISL treatment resulted in suppressed tumor growth, indicating its potential as an adjunct therapy in gastric cancer^142^.

The hydroalcoholic extract of *Ferula assa-foetida*, a traditional medicinal plant, has also been investigated for its anti-cancer effects on GCSCs. This extract exhibited cytotoxic effects on CSCs in a time- and concentration-dependent manner^143^. Treatment with the extract led to a significant decrease in the mRNA levels of EMT markers, such as vimentin, SNAIL1, and ZEB1, as well as the anti-apoptotic factor BCL2 as well as the cell surface markers CD44 and CD54. This suggests that compounds from this plant may inhibit CSC proliferation and induce apoptosis by targeting EMT-related genes and CSC surface molecules, presenting a potential therapeutic avenue for gastric cancer treatment.

7-Difluoromethoxyl-5,4'-di-n-octyl genistein (DFOG), a novel synthetic analogue of genistein, has also been reported to inhibit the stem-like characteristics of GCSCs^144^. DFOG preferentially suppresses self-renewal, migration, and invasion of GCSCs, downregulating the expression of stem cell biomarkers such as CD133, CD44, and aldehyde dehydrogenase 1 (ALDH1). At the molecular level, DFOG decreases the expression of forkhead box M1 (FOXM1) and TWIST1, which are key regulators of CSC stemness and EMT^144^. By modulating these key factors, DFOG reverses the EMT phenotype and reduces the metastatic potential of gastric cancer cells. These findings provide a compelling rationale for the possible future use of DFOG to enhance gastric cancer treatment efficacy^144^.

#### 3.1.2 Traditional Chinese medicinal formulas

Xiaotan Sanjie decoction (XTSJ), a traditional Chinese medicine formula, has demonstrated the ability to attenuate tumor angiogenesis and inhibit the proliferation of GCSCs^145^. *In vitro* studies revealed that XTSJ reduces cell viability and decreases the expression of Notch Homolog 1 (NOTCH1), Hairy and Enhancer of Split 1 (HES1), Vascular Endothelial Growth Factor (VEGF), and Ki-67 in CD44-positive GCSCs. *In vivo* experiments further showed that XTSJ suppresses tumor growth and microvessel density, correlating with reduced expression of CSC markers. The decoction's therapeutic effects are attributed to the inhibition of the Notch-1 signaling pathway, which plays a key role in regulating CSC self-renewal and proliferation^146^.

**Figure 1:**
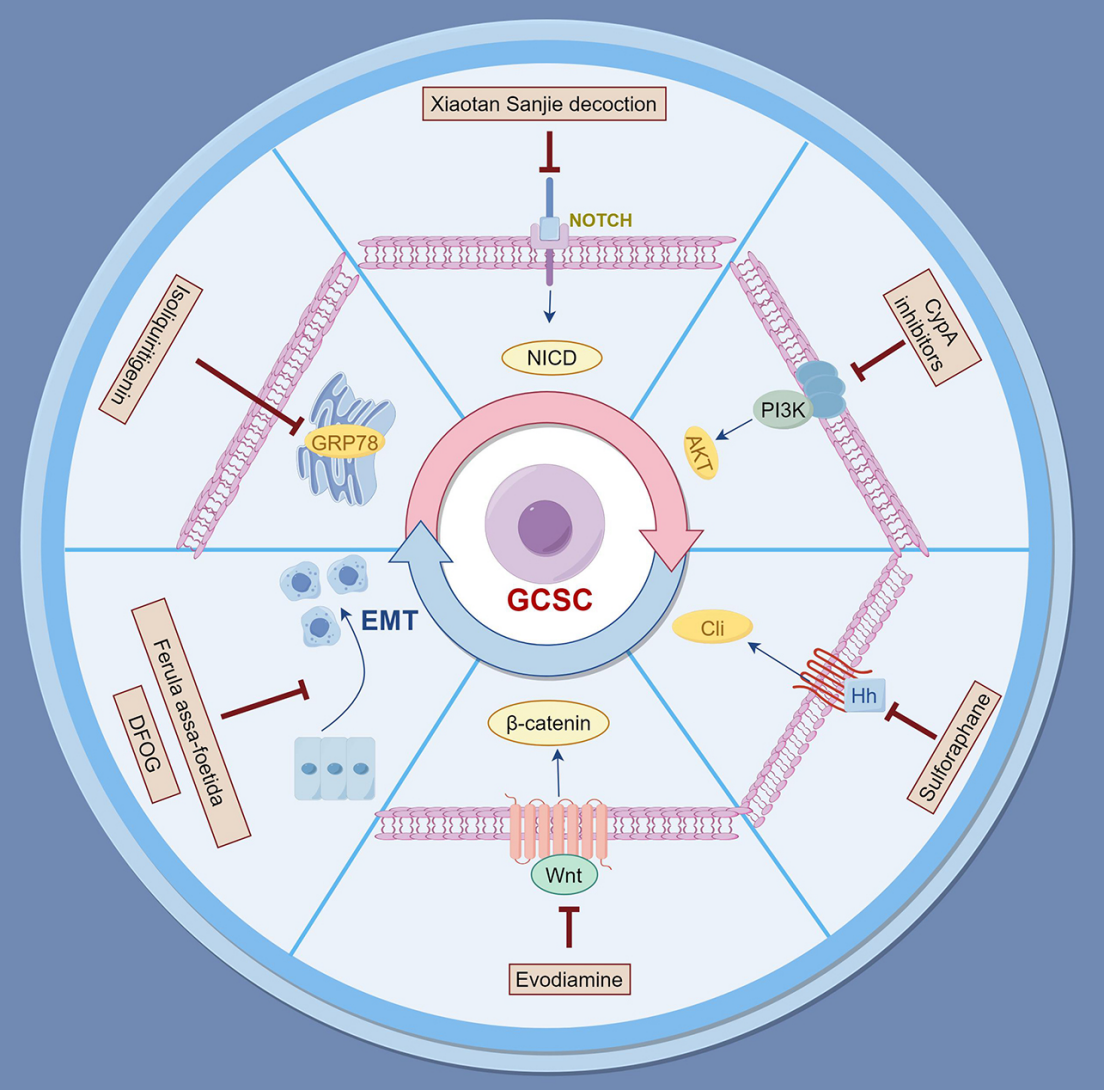
** Natural products for overcoming drug resistance in gastric cancer by targeting cancer stem cells.** The figure depicts key signaling pathways including Wnt/β-catenin, PI3K/AKT, and EMT that are modulated by specific natural compounds.

Natural products provide a rich source of bioactive compounds capable of targeting GCSCs and the overcoming of drug resistance. By modulating critical signaling pathways, such as TGF-β/Smad, NOTCH1, Wnt/β-catenin, and Sonic Hedgehog (SHH), these natural compounds hold great promise for enhancing the efficacy of existing therapies of gastric cancer (Figure 1 and Table 1).

### 3.2 Modulating the tumor microenvironment (TME)

Modulating the TME presents a promising strategy to overcome drug resistance in gastric cancer. The TME comprises various cellular and molecular components, including immune cells, cytokines, ECM elements, and secreted factors which interact with tumor cells to influence cancer progression, metastasis, and response to therapy^147^. Natural products have been explored for their ability to modulate the TME, thereby enhancing the effectiveness of conventional treatments and reversing drug resistance^148^.

#### 3.2.1 Modulation of Tumor-Associated Macrophages (TAMs)

Jianpi Yangzheng Xiaozheng Decoction (JPYZXZ) is an empirical traditional Chinese medicine known for its "Qi-invigorating, spleen-strengthening, and stasis-removing" properties. Research indicates that JPYZXZ improves the quality of life and prolongs survival in gastric cancer patients by affecting TAMs and EMT. *In vitro* studies have demonstrated that JPYZXZ inhibits the motility of gastric cancer cells, reduces tumor cell viability, and decreases the expression of EMT markers such as N-cadherin and Vimentin, while increasing E-cadherin expression^149^. *In vivo*, JPYZXZ treatment led to a significant reduction in tumor weight in mice harboring gastric cancer xenografts. Notably, JPYZXZ was more effective than its individual components at inhibiting EMT transformation. Furthermore, it influenced TAM polarization by promoting the phenotypic switch from M2 (pro-tumor) to M1 (anti-tumor) macrophages, which is crucial for inhibiting tumor growth and metastasis^149^.

Further investigations with a modified Jianpi Yangzheng Decoction (mJPYZ) elucidated its role in inhibiting gastric cancer progression via the macrophage immune checkpoint phosphoinositide 3-kinase gamma (PI3Kγ)^150^. mJPYZ treatment decreased PI3Kγactivity in TAMs, reduced the secretion of the anti-inflammatory cytokine IL-10, and increased the expression of pro-inflammatory cytokines such as TNF-α and interleukin-1 β (IL-1β). This modulation promoted the polarization of TAMs from the M2 to the M1 phenotype, ultimately inhibiting gastric cancer cell EMT and suppressing tumor growth and metastasis. These findings highlight the potential of mJPYZ as a potential therapeutic agent targeting immune components within the TME^150^.

*Dendrobium officinale* polysaccharide (DOP), extracted from the medicinal orchid *Dendrobium officinale*, has demonstrated significant immunomodulatory effects in gastric cancer^151^. *In vitro* studies revealed that DOP can convert M2 macrophages to the M1 phenotype by downregulating the signal transducer and activator of transcription 6 (STAT6)/PPAR-γ and JAGGED1/Notch pathways. This shift in macrophage polarization resulted in decreased expression of the migration-associated proteins N-cadherin and vimentin, while increasing E-cadherin levels, thereby inhibiting gastric cancer cell migration. Additionally, conditioned medium from DOP-treated macrophages promoted apoptosis in gastric cancer cells by upregulating caspase-3 and increasing the BAX/BCL2 ratio. *In vivo*, DOP effectively inhibited tumor growth and reduced Ki-67 levels, indicating decreased cellular proliferation. These results suggest that DOP has the potential to modulate the TME by reprogramming macrophages and inhibiting tumor progression^152^.

#### 3.2.2 Regulation of T lymphocytes

Modified Bu-Zhong-Yi-Qi Decoction (mBYD) is another traditional Chinese medicinal formula that has shown efficacy in prolonging survival in gastric cancer patients as an adjuvant therapy post-chemotherapy^153^. mBYD was found to synergize with the fluoropyrimidine 5-FU, inhibiting gastric cancer progression via modulation of the PD-1/PD-L1-dependent T-cell immune response. In a gastric cancer xenograft model, treatment with mBYD increased the CD4^+^/CD8^+^ T-cell ratio and decreased the proportions of PD-1-expressing CD8^+^ T cells and regulatory T cells (Tregs), thereby enhancing antitumor immunity. Additionally, mBYD inhibited PD-L1 expression through the PI3K/AKT pathway in gastric cancer cells. These immunomodulatory effects contribute to suppressing immune escape by tumors and highlight the potential of mBYD as a potential complementary therapy in gastric cancer^153^.

Oleanolic acid (OA), a pentacyclic triterpenoid known for its anticancer activities, has been found to regulate the balance between Tregs and T helper 17 (Th17) cells in gastric cancer by IL-6 via miR-98-5p^154^. Treatment with OA increased miR-98-5p expression, directly downregulating IL-6 levels. This modulation resulted in a rebalancing of Treg and Th17 cell populations, promoting antitumor immunity. By influencing this critical aspect of the immune response, OA demonstrates significant potential as a therapeutic agent in the possible management of gastric cancer^154^.

#### 3.2.3 Enhancement of Natural Killer (NK) cells and Cytotoxic T Lymphocytes (CTLs)

*Salvia miltiorrhiza* Bunge, commonly known as Danshen, is a traditional Chinese medicinal herb renowned for its cardiovascular benefits and immunomodulatory properties^155^. A neutral polysaccharide fraction extracted from *Salvia miltiorrhiza* (SMPA) has shown significant immune-enhancing effects in gastric cancer models *in vivo^156^*. In a study involving a mutagen N-methyl-N'-nitro-nitrosoguanidine (MNNG)-induced gastric cancer in rats, SMPA treatment resulted in increased body weight and improved immune organ indices. Immunological assessments revealed that SMPA stimulated splenocyte proliferation and modulated cytokine production by promoting anti-inflammatory cytokines such as IL-2, IL-4, and IL-10, while inhibiting pro-inflammatory cytokines like IL-6 and TNF-α. Furthermore, SMPA enhanced the cytotoxic activities of NK cells and CTLs and increased macrophage phagocytic function. These immunomodulatory effects suggest that SMPA could potentially serve as an effective adjunct therapy to enhance immune activity in gastric cancer patients^156^.

#### 3.2.4 Targeting Myeloid-Derived Suppressor Cells (MDSCs)

Curcumin, the principal curcuminoid derived from turmeric (*Curcuma longa*), has been investigated for its ability to modulate the TME by targeting MDSCs^157^. In gastric cancer xenograft models, curcumin treatment inhibited tumor growth and reduced MDSC accumulation in the spleen, blood, and tumor tissues. Curcumin decreased IL-6 levels and inhibited the activation of STAT3 and NF-κB signaling pathways in MDSCs. Furthermore, curcumin promoted the differentiation of MDSCs toward an M1-like phenotype, characterized by increased expression of C-C chemokine receptor type 7 (CCR7) and decreased expression of dectin-1. By inhibiting MDSC-mediated immunosuppression and disrupting their interaction with cancer cells, curcumin enhances antitumor immunity and offers a potential strategy for cancer prevention and therapy^158^.

*Trametes robiniophila Murr* (TRM), a traditional Chinese medicinal fungus, has been used clinically to enhance immunity and improve chemotherapy efficacy. The n-butanol extract of TRM (TRMBE), containing its major bioactive components, has been shown to significantly enhance the anticancer activity of 5-FU in gastric cancer treatment^159^. *In vivo* studies demonstrated that the combination of TRMBE and 5-FU prolonged the survival of mice bearing human gastric cancer xenografts and decreased the risk of liver metastasis^159^. Mechanistically, this combination therapy modulated the TME by reducing immunosuppressive cytokines such as IL-6, IL-10, and TGF-β, while increasing IFN-γ levels in peripheral blood. Additionally, it decreased the levels of polymorphonuclear myeloid-derived suppressor cells (PMN-MDSCs) and PD-1-positive CD8^+^ T cells, while increasing NK cell populations within the TME. These findings suggest that TRMBE enhances the immune response against gastric cancer, thereby enhancing the chemosensitivity to 5-FU and presenting a promising approach for gastric cancer treatment^159^.

Effective modulation of the TME is crucial for enhancing therapeutic outcomes in gastric cancer. Various natural products and traditional Chinese medicinal compounds have demonstrated significant capabilities to influence immune cell dynamics within the TME. This includes the reprogramming of TAMs, enhancing T lymphocyte activity, and activating NK cells and CTLs. Specific formulations such as Jianpi Yangzheng Xiaozheng Decoction, modified Jianpi Yangzheng Decoction, and OA show promise in regulating immune responses that promote antitumor effects and combat immune evasion^160^-^162^. Furthermore, targeting MDSCs and modulating Toll-like receptor signaling pathways underscore the multifaceted roles of these natural agents in overcoming drug resistance. Collectively, integrating these natural products into potential therapeutic strategies for gastric cancer warrants further research and clinical trials to fully realize their potential.

#### 3.2.5 Activation of killer T cells and NK cells

Lentinan, a β-(1,3)-glucan with β-(1,6) branches purified from *Lentinula edodes* (Shiitake mushroom), has been approved in Japan as a biological response modifier for gastric cancer treatment^163^. Clinical studies indicate that when used in combination with chemotherapy, lentinan prolongs survival in patients with advanced gastric cancer compared to chemotherapy alone. Lentinan enhances the cytotoxicity of immune effector cells, including killer T cells and NK cells. *In vitro* studies demonstrated that lentinan increases the cytotoxicity of human PBMCs against tumor cells by inducing the activation of NK cells characterized by CD2^+^, CD16^+^, and CD56^+^ markers. Furthermore, lentinan may synergize with Trastuzumab, a monoclonal antibody targeting the HER2/neu growth factor receptor, which plays a crucial role in tumorigenesis and tumor progression. This synergy activates the complement system through antibody-dependent cellular cytotoxicity and complement-dependent cytotoxicity. These immunomodulatory actions underscore lentinan's potential to improve immune function and clinical outcomes in gastric cancer therapy^163^.

#### 3.2.6 Modulation of toll-like receptor signaling pathways

**Figure 2:**
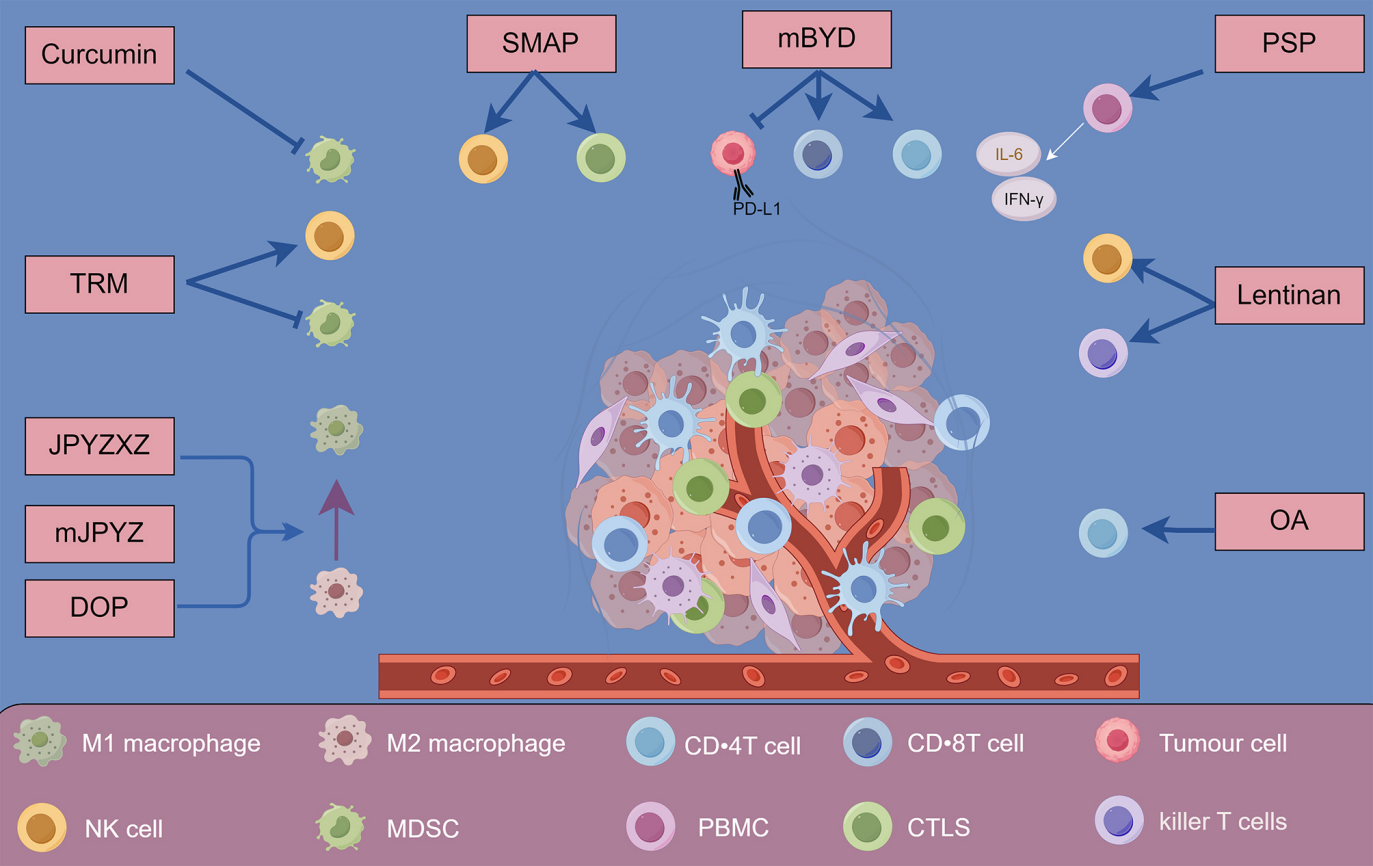
**Natural products for overcoming drug resistance in gastric cancer by modulating the tumor microenvironment.** The figure illustrates how natural products reprogram the immunosuppressive TME in gastric cancer by targeting key cellular components: converting pro-tumor M2 macrophages to anti-tumor M1 phenotype, enhancing T-cell immunity, activating NK/CTL, suppressing MDSCs.

Polysaccharopeptide (PSP), extracted from the medicinal mushroom *Coriolus versicolor*, exhibits significant immunomodulatory properties^164^. PSP has been shown to regulate gene expression and cytokine secretion related to Toll-like receptor (TLR) signaling pathways in human peripheral blood mononuclear cells (PBMCs). Treatment with PSP upregulated the expression of key genes such as IFN-γ, chemokine (C-X-C motif) ligand 10 (CXCL10), TLR4, and TLR5, while downregulating others like TLR9 and TLR10. Additionally, PSP increased the secretion of cytokines including granulocyte colony-stimulating factor (G-CSF), granulocyte-macrophage colony-stimulating factor (GM-CSF), IL-1α, IL-6, and IFN-γ. By enhancing the expression of key molecules in the TLR signaling pathway, particularly the TRAM-TRIF-TNF receptor-associated factor 6 (TRAF6) axis, PSP modulates immune responses and may provide therapeutic benefits in gastric cancer by improving antitumor immunity^165^ (Figure 2 and Table 2).

### 3.3 Exploring natural products as chemosensitizers

Chemoresistance poses a significant obstacle in the treatment of gastric cancer, often leading to poor prognosis and limited efficacy of standard chemotherapeutic agents such as 5-FU, cisplatin, and doxorubicin. Natural products have garnered attention for their potential to sensitize cancer cells to chemotherapy, thereby overcoming drug resistance and enhancing treatment outcomes^166^. Multiple studies have identified various natural compounds exhibiting chemosensitizing effects through mechanisms such as modulation of apoptosis, cell cycle arrest, inhibition of drug resistance-related proteins, metabolic regulation, multidrug resistance efflux pump inhibition and reversal of EMT^51^, ^167^, ^168^.

#### 3.3.1 Modulation of apoptosis and cell cycle arrest

Luteolin, a flavonoid found in many fruits and vegetables, has demonstrated anti-proliferative and chemosensitizing effects on human AGS gastric cancer cells^169^. It inhibits cell growth in a dose- and time-dependent manner and induces G_2_/M cell cycle arrest by downregulating CDC2, CCNB1, CDC25C. Additionally, luteolin promotes apoptosis by increasing pro-apoptotic proteins such as caspases-3, -6, -9, BAX, and p53, and decreasing the levels of the anti-apoptotic protein BCL2, increasing the BAX/BCL2 ratio, hence favoring apoptosis. Notably, luteolin enhances the inhibitory effect of cisplatin when used in combination, indicating its potential as a chemosensitizer in gastric cancer therapy^169^.

Corosolic acid, extracted from *Lagerstroemia speciosa*, has been shown to reduce 5-FU resistance in human gastric cancer cells by activating adenosine monophosphate-activated protein kinase (AMPK)^170^. AMPK is a central regulator of cellular energy homeostasis; elevated AMP:ATP ratio, a consequence of diminished glucose levels, activates AMPK while concurrently repressing mTOR and HIF-1α^171^. This consequently activates glucose and fatty acid uptake and their oxidation. It was found that activation of AMPK leads to decreased expression of thymidylate synthase (TS) and inhibition of the mTOR/4EBP1 signaling pathway. Corosolic acid induces apoptosis and significantly increases the apoptotic cell population when combined with 5-FU, highlighting its therapeutic potential in sensitizing gastric cancer cells to 5-FU through AMPK activation^170^.

#### 3.3.2 Inhibition of drug resistance-related proteins

Gambogic acid (GA), the main active component of gamboge, extracted from gamboge tree (genus *Garcinia*), exhibits potent antitumor effects and has been found to enhance the efficacy of chemotherapeutic agents such as 5-FU, oxaliplatin, and docetaxel against gastric cancer cells^172^. GA synergistically induces apoptosis and downregulates the expression of drug resistance-related genes, including TS, excision repair cross-complementation group 1 (ERCC1), BRCA1, tau, and β-tubulin III. By suppressing these genes, GA enhances chemosensitivity and may serve as an effective adjunct in future gastric cancer treatment^172^.

DT-13, a saponin derived from the flowering plant *Liriope muscari*, has demonstrated the ability to potentiate the sensitivity of gastric cancer cells to topotecan (TPT), a topoisomerase I inhibitor, both in *vitro* and* in vivo*^173^. DT-13 enhances the anti-proliferative effects of TPT by inducing cell cycle arrest and promoting apoptosis. This combination allows for reduced doses of TPT, potentially minimizing its toxicity while maintaining efficacy, offering a promising strategy to improve therapeutic outcomes^173^.

#### 3.3.3 Metabolic regulation and glycolysis inhibition

Catechin, a natural flavonoid found in green tea, has been shown to sensitize 5-FU-resistant gastric cancer cells to 5-FU^174^. Resistant cells exhibit higher lactate production and elevated expression of glycolysis-related enzymes like lactate dehydrogenase A (LDHA). Catechin inhibits LDHA activity and lactate production, thereby limiting glycolysis. The combination of catechin and 5-FU induces reactive oxygen species (ROS)-mediated apoptosis, suggesting that catechin can effectively reduce chemoresistance by targeting LDHA and disrupting cancer metabolism^174^.

#### 3.3.4 Reversal of epithelial-mesenchymal transition

Resveratrol, a polyphenolic compound found in grapes and berries, has been shown to reverse doxorubicin (DOX) resistance in gastric cancer cells^175^. DOX-resistant cells exhibit enhanced migratory capabilities and undergo EMT through AKT activation. Resveratrol inhibits the AKT pathway by activating PTEN, leading to reversal of EMT. This process reduces cell migration and invasion, promotes apoptosis, and sensitizes resistant cells to DOX. The combination of resveratrol and DOX demonstrates a promising approach to surmount chemoresistance in gastric cancer^175^.

**Figure 3:**
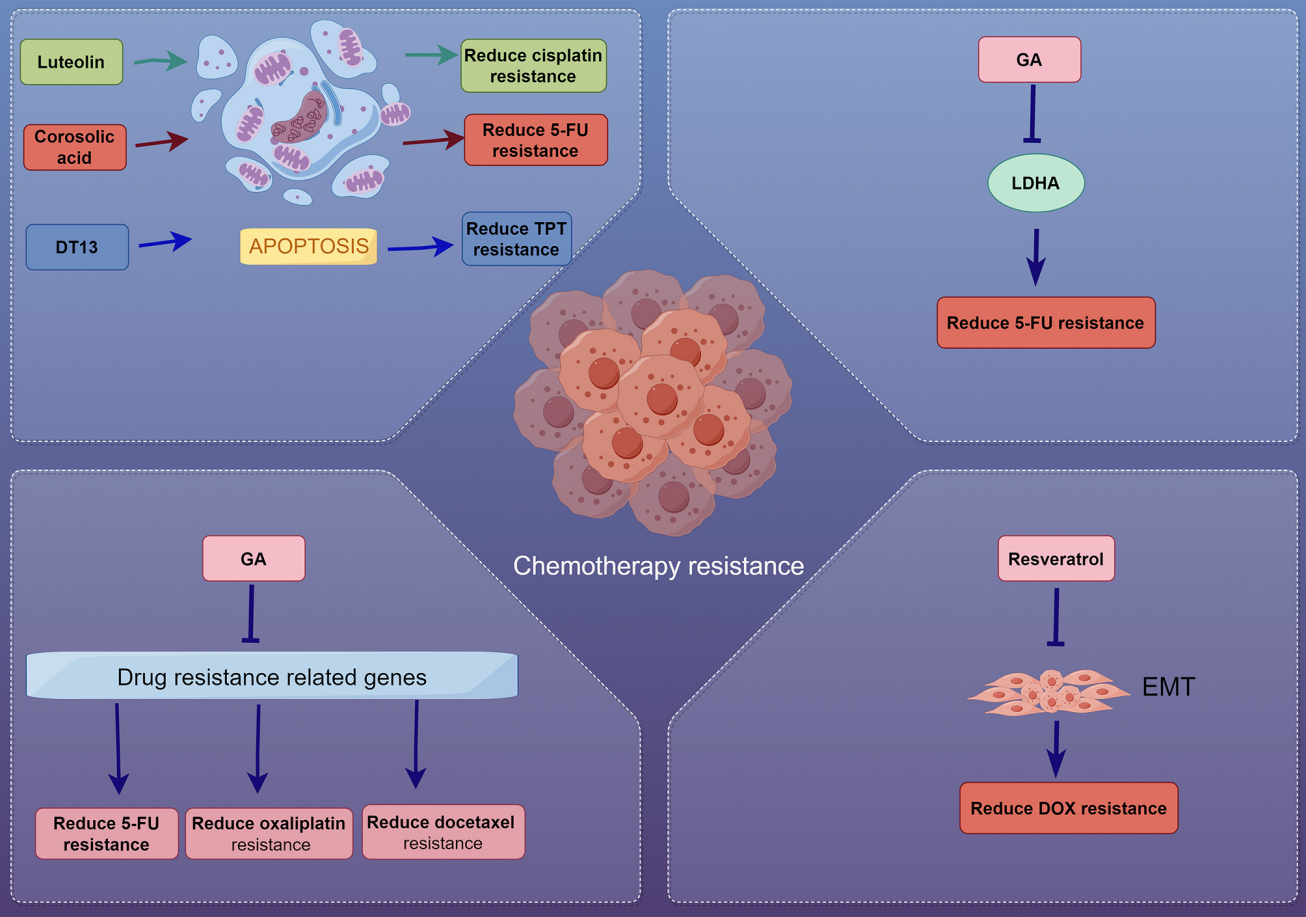
**Exploring natural products as chemosensitizers in gastric cancer.** The figure illustrates how natural products act as chemosensitizers in gastric cancer by modulating drug resistance mechanisms, including inducing apoptosis, inhibiting cell proliferation, downregulating resistance-related proteins, and reversing EMT.

Natural products present a promising avenue for overcoming drug resistance in gastric cancer. Their capacity to modulate apoptosis, influence cell cycle dynamics, inhibit drug-resistant proteins and efflux transporters, and target specific signaling pathways enhances their potential to act synergistically with established chemotherapeutics^176^-^179^. Integrating these natural compounds into treatment regimens can significantly improve outcomes for patients with gastric cancer, paving the way for more effective and less toxic therapeutic strategies (Figure 3 and Table 3).

While natural products demonstrate remarkable potential in overcoming various mechanisms of drug resistance in gastric cancer, their integration with conventional treatment modalities could further maximize therapeutic benefits.

## 4. Combination Therapy: Natural Products Combined with Conventional Chemotherapy and immunotherapy

The intricate interplay between gastric cancer cells and their microenvironment, along with the factors contributing to chemoresistance, underscores the necessity for innovative treatment modalities. As discussed in the preceding sections, the limitations of conventional chemotherapy alone are evident, particularly in the context of resistant tumor phenotypes and the dynamic TME. To address these challenges, a more holistic strategy is warranted, one that integrates natural products with conventional treatment regimens. Natural products not only possess unique biological activities capable of enhancing the efficacy of conventional therapies but also offer potential benefits in modulating tumor characteristics and overcoming distinct mechanisms of drug resistance.

### 4.1 Synergistic effects of natural products with conventional chemotherapy

Combination therapy utilizing natural products has shown significant promise in overcoming drug resistance in gastric cancer^180^. By enhancing the efficacy of conventional chemotherapeutics, these natural compounds can possibly improve patient outcomes. Several studies have elucidated the potential of various natural products in combination with standard chemotherapeutic agents, demonstrating synergistic effects, modulation of apoptosis, and inhibition of drug resistance-related pathways^181^.

#### 4.1.1 5-FU-based combinations

Protocatechuic acid (PCA) is a major metabolite of polyphenols found in green tea displaying a antioxidant and inflammatory activities^182^. When combined with 5-FU, PCA has demonstrated significant antiproliferative effects against human AGS gastric cancer cells^183^. Experiments reveal that PCA, either alone or in combination with 5-FU, inhibits cancer cell proliferation and colony formation while promoting apoptosis^183^. The synergistic effect between PCA and 5-FU is attributed to the upregulation of p53 gene expression and downregulation of the anti-apoptotic protein BCL2. These results indicate that the combination of PCA and 5-FU enhances pro-apoptotic effects, may potentially allow for the reduction in chemotherapeutic dosage; thus, highlighting PCA's potential as a therapeutic adjunct in gastric cancer treatment is warranted^184^.

Kuwanon-A (KA), derived from *Morus alba*, the white mulberry tree, has also been evaluated for its anticancer effects in combination with 5-FU against gastric cancer. This combination therapy induces cell cycle arrest at the G_2_/M phase and promotes apoptosis by activating GADD153 through ER stress signaling pathways. The synergistic anticancer effects of KA and 5-FU were evident both in *vitro* and* in vivo*, suggesting that this combination could possibly serve as an effective regimen for combating gastric cancer^184^.

Curcumin, a polyphenolic compound found in turmeric, has been investigated for its ability to reverse 5-FU resistance in gastric cancer cells by targeting the NF-κB survival-signaling pathway. In a 5-FU-resistant gastric cancer cell line, curcumin effectively inhibited NF-κB activation and reduced the proliferation of 5-FU-resistant cells. Furthermore, the combination of curcumin and 5-FU led to a synergistic increase in apoptosis, establishing curcumin as a potential adjunct therapy to enhance the efficacy of 5-FU in treating gastric cancer^181^.

#### 4.1.2 Cisplatin-based combinations

Paris saponin I (PSI), derived from *Paris polyphylla*, a flowering plant indigenous to China, has shown promise as a chemosensitizer for gastric cancer cells when combined with cisplatin. Studies indicate that PSI sensitizes gastric cancer cell lines to cisplatin while exhibiting low toxicity^185^. Treatment with PSI resulted in a significant reduction in the IC_50_ value of cisplatin in SGC7901 gastric cancer cells. The mechanism underlying this sensitization involves a G_2_/M phase cell cycle arrest and the induction of apoptosis, characterized by decreased expression of BCL2 and increased levels of pro-apoptotic proteins such as BAX and caspase-3. This suggests that PSI could possibly enhance the efficacy of cisplatin in gastric cancer treatment^185^.

Oridonin, a natural diterpenoid derived from the Traditional Chinese herbal medicine plant *Rabdosia rubescens (Isodon rubescens)*, has been identified as a potent agent for reversing cisplatin resistance in human gastric cancer cells^186^. In cisplatin-resistant SGC7901/DDP cells, oridonin significantly inhibited cell proliferation and growth, while effectively decreasing the expression of the key multidrug-resistant efflux transporters P-glycoprotein (P-gp/ABCB1) and multidrug resistance-associated protein 1 (MRP1/ABCC1). Furthermore, oridonin enhanced caspase-dependent apoptosis and demonstrated a synergistic effect when combined with cisplatin, indicating its potential as a novel treatment option for overcoming multidrug resistance in gastric cancer^186^.

#### 4.1.3 Docetaxel-based combinations

Diallyl trisulfide (DATS), derived from garlic, has been shown to enhance chemosensitivity of human gastric cancer cells when combined with the taxene docetaxel^187^. DATS achieves this effect by epigenetically upregulating metallothionein 2A (MT2A), which plays a crucial role in reducing NF-κB activation. The combination of DATS and docetaxel results in a G_2_/M cell cycle arrest and apoptosis, highlighting the synergistic anticancer effects of this natural herbal product. Furthermore, these findings suggest that MT2A may possibly serve as a valuable biomarker for chemosensitivity in patients undergoing docetaxel-based therapy^187^.

#### 4.1.4 Multidrug resistance modulators

Tunicamycin, known for its potent inhibition of *N*-linked glycosylation of proteins, has been evaluated for its ability to reverse multidrug resistance in gastric cancer cells. Research demonstrates that multidrug-resistant gastric cancer cells exhibit increased sensitivity to tunicamycin-induced cell death, largely due to enhanced ER stress^188^. Tunicamycin significantly increases chemotherapy-induced apoptosis in these cells by promoting an ER stress response and triggering autophagy. This mechanism enhances the effects of chemotherapeutic agents when used in combination, underscoring the potential of targeting *N*-linked glycosylation to overcome chemoresistance in gastric cancer^188^.

Pseudolaric acid B (PAB), a diterpene acid isolated from the pine tree family *Pseudolarix quanbaizhi*, has demonstrated significant anti-tumor activity and the overcoming of the multidrug resistance phenotype in SGC7901/ADR cells^189^. PAB not only inhibited cell proliferation and induced apoptosis but also diminished P-gp and cyclooxygenase-2 (COX2) expression. When combined with conventional chemotherapeutic agents, PAB exhibited enhanced effects on inhibition of cell growth, suggesting that it can enhance the efficacy of chemotherapy while also restoring sensitivity in drug resistant gastric cancer cells^189^.

The synergy between natural products and conventional chemotherapy offers a promising approach to enhance treatment efficacy in gastric cancer. Various natural compounds have shown significant potential when combined with standard chemotherapeutics such as 5-FU, cisplatin, and docetaxel^187^-^189^. These natural products enhance apoptosis, inhibit drug-resistant pathways, thereby offer possible opportunities to reduce the doses of conventional drugs, potentially minimizing their side effects. Such findings underscore the importance of possibly integrating natural products into treatment regimens, as they can improve patient outcomes by overcoming chemoresistance and enhance the efficacy of existing therapies (Table 4).

### 4.2 Synergistic effects of natural products with immunotherapy

The integration of natural products with immunotherapy represents a promising strategy for enhancing the effectiveness of gastric cancer treatments. Recent studies have highlighted various natural compounds that can modulate immune responses and improve the efficacy of immunotherapeutic agents.

#### 4.2.1 Enhancing immunotherapy through epigenetic modulation

OA is a natural compound that has shown promise in enhancing the efficacy of immunotherapy through epigenetic modulation^190^. In gastric cancer cells, OA was found to downregulate PD-L1 expression by inhibiting the NF-κB signaling pathway and promoting DNA demethylation. Co-culture experiments demonstrated that OA treatment restored IL-2 levels and enhanced T cell-mediated cytotoxicity against gastric cancer cells. Notably, the presence of PD-L1 diminished the cytotoxic activity of T cells; however, PD-1 blocking antibodies were able to restore T cell activity, indicating the importance of PD-L1 modulation in OA's mechanism of action^190^.

#### 4.2.2 Modulating immune responses

mBYD has demonstrated significant potential as an adjunct therapy to 5-FU, particularly in terms of its effects on T cell activation via the PD-1 pathway^191^. In a comprehensive study employing a gastric cancer xenograft model, the combination of mBYD and 5-FU significantly prolonged mice survival compared to either treatment alone. Flow cytometry analyses revealed an increase in the CD4^+^/CD8^+^ T cell ratio in the mBYD group, accompanied by a reduction in CD8^+^PD-1^+^ T cells and PD-1^+^ T regulatory cells. Mechanistically, mBYD was found to inhibit PD-L1 expression through the PI3K/AKT signaling pathway, thereby promoting T lymphocyte proliferation, activation, and cytotoxic activity. These findings suggest that mBYD effectively modulates peripheral immunity and counteracts immune evasion in gastric cancer.

#### 4.2.3 Regulating MicroRNA for enhanced immune response

Additionally, astragaloside IV (AS-IV) is a major compound isolated from an aqueous root extract of the leguminous herb *Astragalus membranaceus*. AS-IV is a cycloartane-type triterpene glycoside, which was found to regulate microRNA expression, particularly miR-195-5p, which targets PD-L1^192^. AS-IV was shown to inhibit EMT and angiogenesis in gastric cancer cells by upregulating miR-195-5p, leading to decreased PD-L1 levels. This mechanism highlights AS-IV's potential not only to suppress the aggressiveness of cancer cells but also to enhance the immune response against gastric cancer^192^.

#### 4.2.4 Multi-target regulation of PD-L1

Finally, Banxia Xiexin decoction (BXXX) has been found to inhibit PD-L1 expression through a multi-target and multi-pathway approach^193^. BXXX effectively reduced the expression of key oncogenes and regulated various signaling pathways associated with PD-L1 in gastric cancer. This regulatory mechanism led to decreased gastric cancer cell proliferation and increased apoptosis, suggesting that BXXX may serve as a valuable therapeutic option in combination with immunotherapy^193^.

These studies collectively illustrate the potential of natural products to synergize with immunotherapy by enhancing immune responses, regulating immune checkpoints, and favorably modifying the TME (Table 5).

### 4.3 Case studies and clinical trials

The combination of natural products with conventional therapies has been investigated in various clinical settings, demonstrating promising results in enhancing treatment efficacy against gastric cancer^194^. This section presents selected case studies and clinical trials that illustrate the potential benefits of integrating natural products into cancer treatment regimens.

#### 4.3.1 *Rhus verniciflua* stokes extract in an elderly patient

An 82-year-old female with gastric adenocarcinoma was treated with standardized *Rhus verniciflua* Stokes (RVS) extract (900 mg/day). After five months, gastroscopy and CT scans demonstrated significant tumor mass reduction. The patient remained well 29 months post-treatment, suggesting RVS's potential as a natural therapeutic agent^195^,^196^.

#### 4.3.2 Garlic supplementation reduces gastric cancer mortality

A long-term randomized controlled trial in China found that garlic supplementation for 7.3 years significantly lowered gastric cancer mortality, with protective effects becoming evident after approximately 12 years and sustained over 22.3 years, indicating garlic as a safe, cost-effective preventive option^194^.

#### 4.3.3 *Marsdenia tenacissima extract* (MTE)as an adjuvant therapy

A systematic review of 17 randomized trials with 1,329 patients showed that MTE enhanced chemotherapy response rates, improved patient performance status, and reduced some chemotherapy-related side effects. It also suggested improvement in progression-free and overall survival within certain trials, supporting MTE's role as an adjunct therapy^197^.

#### 4.3.4 Curcumin for chemoprevention in high-risk patients

A phase I trial administering oral curcumin up to 8,000 mg/day to patients with premalignant lesions demonstrated safety and some histological lesion improvements, although malignancies developed in a few patients. Curcumin shows promise for chemoprevention pending further studies^198^,^199^.

#### 4.3.5 *Aloe arborescens* in combination with chemotherapy

In a randomized trial of 240 metastatic gastric cancer patients, *Aloe supplementation* alongside chemotherapy significantly improved tumor regression rates and disease control compared to chemotherapy alone, indicating enhanced treatment efficacy^200^.

Collectively, these case studies and clinical trials provide some evidence for the efficacy of possibly integrating natural products with conventional cancer therapies in the future treatment of gastric cancer (Table 6).

## 5. Future Directions and Research Opportunities

While the preclinical studies and some clinical observations suggest the potential of natural products in enhancing gastric cancer therapy, several challenges need to be addressed before their widespread clinical adoption.

These include the need for rigorous clinical trials to confirm efficacy and safety, standardization of natural product formulations, and a deeper understanding of their mechanisms of action and interactions with conventional therapies. Addressing these gaps will pave the way for the rational integration of natural products into evidence-based gastric cancer treatment strategies.

### 5.1 Need for clinical trials

Integrating natural products into gastric cancer therapy shows promise in enhancing efficacy and overcoming drug resistance, but rigorous clinical trials are essential to validate their safety and effectiveness. Clinical trials help evaluate the pharmacokinetics, pharmacodynamics, and interactions of natural products with standard therapies like 5-FU and cisplatin, optimizing regimens and minimizing adverse effects. They also facilitate the identification of biomarkers, such as HER2 and PD-L1, to predict which patients benefit most from natural product-based treatments.

Long-term follow-up in trials is critical for assessing the durability of responses, effects on survival, and impacts on tumor recurrence and metastasis. As natural products often have multifaceted mechanisms, understanding their long-term effects is key to defining their role in managing gastric cancer.

Comprehensive clinical trials are vital to translating preclinical findings into effective therapies and developing innovative strategies to improve outcomes in this challenging disease.

### 5.2 Exploration of new natural compounds

Exploring new natural compounds is vital for addressing drug resistance in gastric cancer. Advances in phytochemistry and molecular biology have led to the discovery of diverse natural products from plants, fungi, and marine organisms with unique anticancer mechanisms that complement existing therapies. Compounds like curcumin show promise in modulating pathways related to cell proliferation, apoptosis, and drug resistance.

Systematic screening and characterization, including high-throughput methods, are crucial for identifying candidates and evaluating their efficacy against specific resistance pathways. Integrating technologies such as genomics, transcriptomics, and metabolomics can further elucidate the molecular targets and mechanisms of these compounds, optimizing their combination with chemotherapy or immunotherapy.

Collaboration between traditional medicine and modern research can facilitate the discovery of novel bioactive compounds. Traditional medicinal knowledge, when scientifically validated, has the potential to uncover innovative therapies for gastric cancer, offering new opportunities for overcoming chemoresistance. However, as we advance in the exploration and application of these natural compounds, it becomes increasingly important to comprehensively evaluate their complex pharmacological profiles and potential therapeutic implications.

To fully harness the potential of natural compounds while minimizing potential risks, future research should prioritize the following aspects. The multi-target characteristics of natural products constitute a double-edged sword. On one hand, natural compounds can simultaneously modulate multiple signaling pathways, thereby effectively overcoming single-target resistance mechanisms. This broad bioactivity permits natural products to address complex resistance phenotypes in gastric cancer, including apoptosis evasion, enhanced DNA repair, and tumor microenvironment remodeling. On the other hand, promiscuous targeting may lead to unintended off-target interactions or dose-dependent toxicity. For example, curcumin's inhibition of NF-κB has been widely reported to sensitize resistant cells to chemotherapy; yet at high concentrations, curcumin can inhibit cytochrome P450 enzymes and affect the metabolism of co-administered chemotherapeutics, potentially reducing drug clearance or increasing systemic toxicity^201^. Consequently, there is an urgent need to elucidate the dose-response relationship of each natural product, to define a therapeutic window that maximizes synergistic anticancer effects while minimizing adverse reactions. Advanced pharmacokinetic and pharmacodynamic studies-together with systems pharmacology modeling-are warranted to predict and monitor off-target liabilities. Moreover, rational formulation strategies, such as controlled-release nanoparticles or prodrug approaches, may further optimize tissue selectivity. Only through a balanced, dialectical evaluation of multi-target benefits versus potential risks can natural products be safely and effectively integrated into combination regimens for gastric cancer.

### 5.3 Synergistic potential of natural products with small molecule inhibitors

While this review has primarily focused on natural products' role in overcoming chemotherapy resistance, it is crucial to acknowledge that current first-line treatments for gastric cancer increasingly rely on small molecule inhibitors targeting specific oncogenic pathways. However, the therapeutic potential of these small molecule inhibitors is frequently constrained by acquired resistance mechanisms, limited bioavailability, and dose-limiting toxicities, creating an urgent need for innovative combinatorial approaches.

The integration of natural products with small molecule inhibitors represents a promising strategy to enhance therapeutic efficacy while potentially mitigating resistance development. Natural products offer several advantages in this context: their multi-target mechanisms can simultaneously modulate multiple resistance pathways, their generally favorable safety profiles allow for prolonged administration, and their diverse chemical scaffolds provide opportunities to overcome single-target resistance mechanisms that commonly limit small molecule inhibitors. However, the combination of natural products with small molecule inhibitors also presents potential risks that warrant careful evaluation. Competitive inhibition may occur when natural products interfere with inhibitor metabolism or transport.

The strategic combination of natural products with small molecule inhibitors represents a paradigm shift toward more holistic, multi-targeted therapeutic approaches in gastric cancer. By leveraging the complementary strengths of both therapeutic modalities while carefully managing potential interactions, this strategy holds significant promise for improving treatment outcomes and overcoming the persistent challenge of drug resistance in gastric cancer management.

### 5.4 Personalized medicine approaches

Advancements in genomic and proteomic technologies have identified crucial biomarkers, such as HER2, PD-L1, and MSI, which guide personalized therapies in gastric cancer. Incorporating these biomarkers into clinical decisions allows oncologists to predict treatment responses and tailor regimens, including combining chemotherapy with natural products. Pharmacogenomics further refines personalized strategies by evaluating genetic variations in drug metabolism, like cytochrome P450, enabling precise dosing of conventional drugs and natural compounds to improve outcomes and reduce toxicity.

Natural products bring new opportunities for overcoming drug resistance due to their multitargeted actions. By leveraging tumor-specific molecular profiles, compounds targeting pathways like cell survival and apoptosis can enhance therapeutic success. Combination therapies in personalized medicine are promising, as pairing natural products with standard chemotherapeutics can improve response rates and reduce resistance development. Ultimately, integrating natural products into personalized medicine represents a transformative approach to combat gastric cancer effectively.

## 6. Conclusions

**Figure 4:**
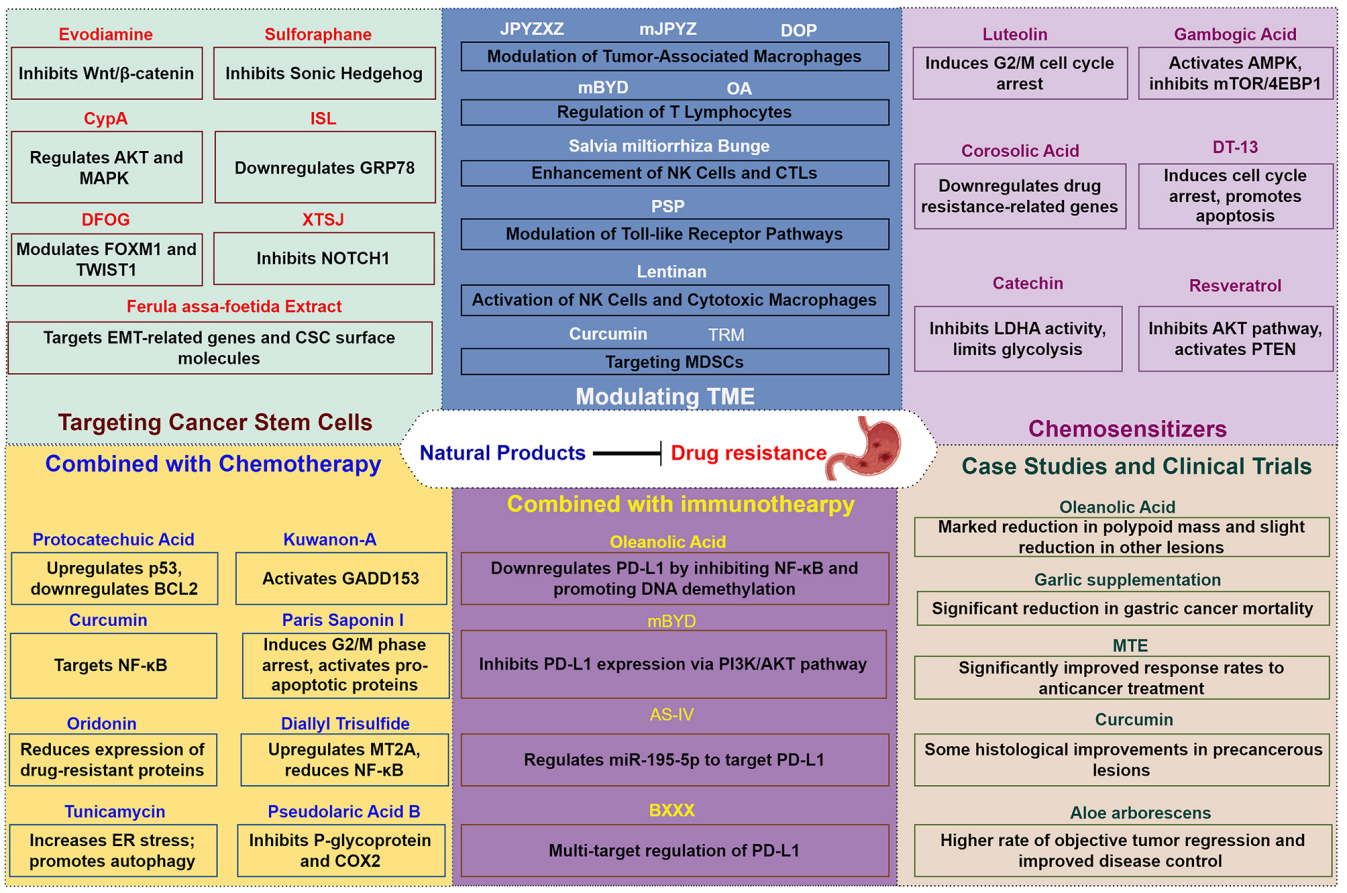
** Strategies for overcoming drug resistance in gastric cancer using natural products.** this schematic illustrates the mechanisms of action of various natural products in targeting CSCs, modulating the TME, and acting as chemosensitizers in gastric cancer. The figure also highlights examples of combination therapy approaches using natural products with conventional chemotherapy and immunotherapy, along with case studies and clinical trial results demonstrating their efficacy in diminishing drug resistance and improving clinical outcomes.

Drug resistance poses a significant challenge in gastric cancer treatment, necessitating innovative strategies. Natural products have emerged as promising agents to enhance therapeutic efficacy and overcome chemoresistance. These compounds exhibit multifaceted mechanisms, including inducing apoptosis, modulating critical signaling pathways, transforming the TME, and reversing resistance (Figure 4). Notably, their synergistic effects with conventional chemotherapy and immunotherapy offer improved outcomes while reducing toxicity.

Future gastric cancer therapies will benefit from comprehensive clinical trials to validate the safety and efficacy of natural products across diverse populations. Advances in biomarker identification and pharmacogenomics, combined with the integration of traditional medicinal knowledge and modern molecular research, will drive the discovery of novel therapeutic agents. This holistic approach not only enhances existing treatments but also improves overall patient outcomes. Embracing natural products and personalized medicine offers a transformative path for addressing drug resistance in gastric cancer, providing hope for more effective, targeted, and less toxic treatments.

## Figures and Tables

**Table 1:** Summary of natural products targeting CSCs

Natural Product	Mechanism of Action	Effects on GCSCs	References
Evodiamine	Inhibits Wnt/β-catenin signaling pathway	Reduces tumor cell proliferation, induces apoptosis, decreases sphere-forming ability, and suppresses EMT markers.	137
Sulforaphane	Inhibits Sonic Hedgehog (SHH) signaling pathway	Reduces tumor sphere formation, decreases CSC marker expression, induces apoptosis, and suppresses tumor cell proliferation.	139
Cyclophilin A Inhibitors	Regulates CypA/CD147-mediated AKT and MAPK signaling pathways	Suppresses tumor cell proliferation, induces apoptosis, and decreases expression of key CSC markers.	141
Isoliquiritigenin	Downregulates GRP78, an ER chaperone involved in chemoresistance	Inhibits CSC-like properties, reduces stemness-related protein expression, and suppresses tumor growth in xenografts.	142
Ferula assa-foetida Extract	Targets EMT-related genes and CSC surface molecules	Exhibits cytotoxic effects on GCSCs, reduces mRNA expression of EMT markers, and induces apoptosis.	143
7-Difluoromethoxyl-5,4'-di-n-octyl genistein	Modulates FOXM1 and TWIST1, key regulators of CSC stemness and EMT	Inhibits self-renewal, migration, and invasion of GCSCs, downregulates expression of CSC biomarkers.	144
Xiaotan Sanjie Decoction	Inhibits NOTCH1 signaling pathway	Reduces cell viability, decreases expression of CSC biomarkers, and suppresses tumor growth and microvessel density.	146

**Table 2:** Overview of natural products modulating the TME in gastric cancer

Natural Product	Mechanism of Action	Effects on TME	References
Jianpi Yangzheng Xiaozheng Decoction	Modulates TAMs, enhances survival, improves quality of life	Inhibits gastric cancer cell motility, reduces EMT markers, enhances M1 macrophage polarization	149
Modified Jianpi Yangzheng Decoction	Targets PI3Kγin TAMs	Decreases IL-10, increases TNF-α and IL-1β, inhibits gastric cancer cell EMT	150
*Dendrobium officinale* Polysaccharide	Reprograms macrophage phenotype using STAT6 and Notch pathways	Converts M2 macrophages to M1, decreases N-cadherin and Vimentin, inhibits tumor cell migration	151
Modified Bu-Zhong-Yi-Qi Decoction	Enhances PD-1/PD-L1 T-cell immune response	Increases CD4^+^/CD8^+^ T-cell ratio, decreases PD-1 expression, enhances anti-tumor immunity	152
Oleanolic Acid	Regulates Treg and Th17 cell balance via IL-6 modulation	Promotes anti-tumor immunity by rebalancing Treg and Th17 cell populations	153
*Salvia miltiorrhiza* Bunge Polysaccharide	Boosts immune function and cytokine production	Increases NK cell and CTL cytotoxicity, enhances splenocyte proliferation	155
Polysaccharopeptide	Modulates TLR signaling pathways	Enhances expression of IFN-γ, CXCL10; increases cytokine secretion	164
Lentinan	Enhances cytotoxicity of immune effector cells	Improves NK cell activation and prolongs survival in chemotherapy	163
Curcumin	Targets Myeloid-Derived Suppressor Cells (MDSCs)	Reduces MDSC accumulation, inhibits IL-6 and STAT3 signaling, enhances anti-tumor immunity	158
TRM n-butanol Extract	Enhances anticancer activity of chemotherapy	Increases NK cell populations, decreases immunosuppressive cytokines and MDSCs	159

**Table 3:** Overview of natural products and their mechanisms as chemosensitizers in gastric cancer

Natural Product	Mechanism of Action	Effects on Chemoresistance	References
Luteolin	Induces G2/M cell cycle arrest, modulates apoptosis	Inhibits AGS cell growth, enhances cisplatin efficacy	169
Corosolic Acid	Activates AMPK, inhibits mTOR/4EBP1 signaling	Reduces 5-FU resistance, increases apoptotic cell population	170
Gambogic Acid	Downregulates drug resistance-related genes	Enhances efficacy of 5-FU, oxaliplatin, and docetaxel	172
DT-13	Induces cell cycle arrest, promotes apoptosis	Potentiates sensitivity to topotecan, allows reduced dosing	173
Catechin	Inhibits LDHA activity, limits glycolysis	Restores 5-FU sensitivity in5-FU-resistant cancer cells, induces ROS-mediated apoptosis	174
Resveratrol	Inhibits AKT pathway, activates PTEN	Reverses DOX resistance, reduces tumor cell migration and invasion	175

**Table 4:** Overview of natural products combined with conventional chemotherapy in gastric cancer treatment

Natural Product	Conventional Chemotherapy	Mechanism of Action	Effects on Chemoresistance	References
Protocatechuic Acid	5-FU	Upregulates p53, downregulates BCL2	Enhances pro-apoptotic effects, inhibits cell proliferation	182
Kuwanon-A	5-FU	Activates GADD153 via ER stress signaling	Induces G_2_/M phase arrest, promotes apoptosis	184
Curcumin	5-FU	Targets NF-κB survival signaling pathway	Reverses 5-FU resistance, increases apoptosis	181
Paris Saponin I	Cisplatin	Induces G2/M phase arrest, activates pro-apoptotic proteins	Sensitizes cells to cisplatin; decreases IC_50_ in SGC7901 cells	185
Oridonin	Cisplatin	Reduces expression of drug-resistant proteins	Enhances sensitivity to cisplatin, induces apoptosis	186
Diallyl Trisulfide	Docetaxel	Epigenetically upregulates MT2A, reduces NF-κB activation	Induces G_2_/M cell cycle arrest and apoptosis	187
Tunicamycin	Various chemotherapeutics	Increases ER stress; promotes autophagy	Enhances chemotherapy-induced apoptosis in resistant cells	188
Pseudolaric Acid B	Various chemotherapeutics	Inhibits P-glycoprotein and COX2	Restores sensitivity in resistant cells, enhances chemotherapy efficacy	189

**Table 5:** Overview of natural products that synergize with immunotherapy in gastric cancer treatment

Natural Product	Mechanism of Action	Effects on Immunotherapy	References
Oleanolic Acid	Downregulates PD-L1 by inhibiting NF-κB and promoting DNA demethylation	Enhances T cell cytotoxicity, restores IL-2 levels	190
Modified Bu-zhong-yi-qi Decoction	Inhibits PD-L1 expression via PI3K/AKT pathway	Promotes T lymphocyte activation, increases CD4^+^/CD8^+^ T cell ratio	191
Astragaloside IV	Regulates miR-195-5p to target PD-L1	Inhibits EMT and angiogenesis, enhances immune response	192
Banxia Xiexin Decoction	Multi-target regulation of PD-L1	Reduces oncogene expression, increases apoptosis in gastric cancer cells	193

**Table 6:** Clinical evidence of natural product impact on gastric cancer in human populations

Natural Product	Population	Findings	References
*Rhus verniciflua* Stokes extract	An elderly female patient	Marked reduction in polypoid mass and slight reduction in other lesions; patient is alive and well. Suggests potential to induce apoptosis and inhibit growth.	195
Garlic supplementation	3,365 patients	Significant reduction in gastric cancer mortality.	194
*Marsdenia tenacissima* extract	1,329 patients	Significantly improved response rates to anticancer treatment; reduced chemotherapy-related side effects; potential to prolong survival.	197
Curcumin	25 patients	Non-toxic; demonstrated safety and biological effects; some histological improvements in precancerous lesions.	199
*Aloe arborescens*	240 patients	Higher rate of objective tumor regression and improved disease control compared to chemotherapy alone; suggests enhanced efficacy of chemotherapy.	200
